# A Case of Uretroplasty

**Published:** 1876-08

**Authors:** 

**Affiliations:** Munich


### MISCELLANEOUS.

A CASE OF URETEROPLASTY.—By Prof. Nussbaum, of Munich. (Abstract from Bavarian *Intelligenz Blatt*, by Dr. Petershausen.) —Ovariotomy has been performed more than a thousaud times, when it happened that Prof. Simon, of Heidelberg, accidentally cut through the left ureter at an operation. As he was compelled at the same time to remove a large part of the uterus, the urine afterwards ran both through the abdominal fistula, which formed itself in the cicatrix, and through the cervical cavity and through the vagina. Great suffering was caused to the patient by this state of things. But at last the professor ended her miserable condition by resorting to renotomy, which operation was a complete success, the remaining kidney assuming a two-fold function. Afterwards a similar misfortune occurred to Prof. Nussbaum, while removing an ovarian cyst of fifteen years' standing. The most difficult part of the work was the separation of the tumor from the uterus. Thus far the operation was successful; but it was noticed that, after several days, the quantity of urine was much less than it should have been, while at the same time a watery excretion oozed out of the abdominal wound. A close examination of the excretion showed that it contained urine. As the bladder was not injured, it seemed that the injury resembled that in Simon's case. An examination proved that the right ureter, which was closely connected with the ovary, had been cut during the operation. The woman made a quick recovery, but as there remained an abdominal fistula, through which one-half of the urine escaped, the poor woman was so exceedingly troubled by it that the doctor at last concluded to put an end to her suffering. There were different methods which the doctor had in view for this purpose. In this case he thought it would be best to conduct the urine, excreted by the fistula, to the bladder by the shortest way. This could be done, either by uniting the cut parts of the ureter or by forming an artificial one. The doctor intended to try the first method. The abdominal fistula was dilated for two days by laminaria bougies. Then intruding his left forefinger into the fistula, he could reach a reservoir of the urine, and the termination of that part of the ureter which was connected with the kidney. To reach the other end of the ureter the doctor widened the urethra by Simon's dilator, but he was unsuccessful in finding the termination of the ureter. When he introduced a male catheter into the bladder, and pushing the latter upwards by it, his left forefinger in the fistula could be touched, the intervening space being no greater than inch. This space was now penetrated by a trocar, and in the opening there was introduced a drainage tube. To its lower part there was attached a glass tube with a broad margin, by which an escape of the drainage tube toward the fistula was to be prevented, while a silver wire, fastened to the upper end of the tube, was led from this point through the fistula, and, by strips of adhesive plaster, attached to the skin. The drainage tube, which in this manner was kept in position, was intended for a supplementary ureter. Incontinence followed for three days, but in general the patient got along nicely. Some days later the tube unfortunately escaped, in spite of its rim, through the newly formed ureter, and this became obstructed by flocculent matter. The urine was again excreted by the fistula, and the doctor had to introduce another tube. The upper end of this was likewise connected with a silver wire, but it was so much longer that its lower end protruded from the urethra so that the urine ran into the vessel. The patient was very unwell for some days; but little urine was excreted by the fistula. To the astonishment of the operator he found the drainage tube, two days after its introduction, upon the patient's belly. This tube had also slipped upwards, as in the previous instance. However an injection of litmus solution convinced the doctor that the artificial way was still open, and consequently, he did not introduce another tube. The artificial way kept intact, a normal quantity of urine was excreted, and when, several weeks later a few drops of urine were still excreted by the fistula, which was then very narrow, this was closed by cauterization. The patient's health became excellent afterwards."—*Medical Record.*

